# A Community of Shared Future for Mankind: A study on the news discourse of environmental cooperation in countries along the Belt and Road Initiative

**DOI:** 10.1371/journal.pone.0293296

**Published:** 2023-10-23

**Authors:** Niwen Liu, Megat Al Imran Yasin, Syed Agil Alsagoff, Chwee Fang Ng, Mengyu Li

**Affiliations:** 1 Faculty of Modern Languages & Communication, Universiti Putra Malaysia, Serdang, Malaysia; 2 Faculty of Humanities & Social Sciences, Nanjing Forestry University, Jiangsu, Nanjing, China; University of Pecs Faculty of Humanities: Pecsi Tudomanyegyetem Bolcseszettudomanyi Kar, HUNGARY

## Abstract

‘The Belt and Road Initiative’ (B&R) was proposed by Chinese President Xi Jinping in September and October 2013 and is now actively supported and participated by more than 100 countries and international organizations. B&R has become a hot topic all over the world since its inception. However, the environmental issues arising from this Initiative should not be underestimated. The concept of ‘A Community of Shared Future for Mankind’ is being promoted under the context of globalization, and there has been a lot of coverage in the mainstream media from various countries on the topic of environmental cooperation along B&R. This study takes a sample of reports on the ‘Belt and Road Environmental Cooperation’ from July 2021 to August 2022 and uses Van Dijk’s theory of news discourse analysis to analyze 20 articles in depth. This study attempts to explore the kind of thematic structure and lexical style that the mainstream newspapers from different countries use to report the environmental cooperation among the countries along B&R, also the implications of such a thematic structure and lexical style, and the characteristics of the discourse construction of mainstream newspapers in different countries. The research has found that B&R countries are used to holding a positive attitude to make a report and seek international cooperation. The headlines are mostly made up of nouns, and both direct and indirect quotations are used. Besides, to enhance the truth of the report, different number types are also involved; the theme structures are often made up of a two-level hierarchy.

## 1 Introduction

With the increasing severity of the global ecological crisis, how to effectively protect the ecological environment and achieve sustainable global economic and social development has become a major issue facing mankind. Only if all countries in the world make positive changes and follow a green development path together can human civilization be developed forever. At the National Conference on Ecological Protection, the President of China, Xi Jinping, proposed ‘A Community of Shared Future for Mankind’, and protecting the ecological environment is a common challenge and shared responsibility for the world. This reveals that after a certain stage of development of civilized industrial society, human society is bound to move towards the operation of an ecological community. Global ecological conservation and governance provide an important path for building a community of human destiny, which can reconstruct the relationship between human beings and nature to achieve harmony and unity. Global ecological protection and governance in the context of ‘A Community of Shared Future for Mankind’ is urgently needed to promote global green development and build a global ecological civilization.

Since ‘the Belt and Road Initiative’ (B&R) was proposed, it has received great attention from countries worldwide, especially the mainstream media coverage. The media from a variety of countries have reported on the launch of the initiative, its development, and the problems it may encounter.

Focusing on the "the Belt and Road" and green construction, the academic community has also begun to pay attention to and form relevant achievements. By consulting relevant representative literature, it can be roughly divided into different perspectives, such as the definition of green development connotation, the interpretation of green "the Belt and Road" connotation, the exploration of development status and the exploration of future paths. Some scholars believed that the path of green development is to protect the ecological environment in pursuit of sustainable human development, emphasizing the need for green development and ensuring the coordination and unity of economic development and environmental protection [1–4]. At the same time, the construction of the "the Belt and Road" cannot simply consider the economic benefits along the line and ignore the appeal of regional people to the quality of life, so the green "the Belt and Road" construction is a win-win move. The green economy is a "sustainable economy", and future economic development should reflect affordability. This affordability is determined by the natural environment and human acceptance range, ensuring the rationalization of the entire production and consumption process, and sustainability will not be forced to stop due to blind selection, information lag, and other issues. Foreign scholars also interpreted the green Development theory from the perspective of ecological Marxism, and put forward unique views on the causes and solutions of the ecological environment crisis, which greatly enriched the theoretical system of green development. Most of the countries along B&R are developing countries with low levels of economic development and a serious ecological and environmental situation. Most of them are facing poor environmental foundations, heavy population burdens, weak environmental protection capabilities, and inadequate cooperation systems. However, most studies on the B&R report have focused on comparing the word used and attitudes of the media from different countries. Very few studies have analyzed the environmental issues and cooperation related to B&R.

This study takes Van Dijk’s [5] framework of journalistic discourse analysis as the theoretical framework and uses the news text of B&R environment-related reports from a variety of countries to conduct a systematic and in-depth analysis of the journalistic discourse from three aspects: text, context, and construction of discourse meaning.

On the one hand, this study is a complement to international communication and cooperation research by providing discourse analysis. At the same time, it adds value to communication research by providing a discourse analysis of mainstream newspaper coverage of the environmental cooperation of B&R. On the other hand, this study can effectively promote the linkage of information among various countries along the route and facilitate the development of B&R. At the same time, this study is in line with ‘A Community of Shared Future for Mankind’ and provides a good reference for research on how to report on international cooperation.

## 2 Literature review

The academic community is paying more and more attention to the "the Belt and Road" initiative, and the research field is more and more broad. The specific contents of literature research can be summarized into the following categories: understanding of the connotation of the "the Belt and Road" initiative, research on the significance of the "the Belt and Road" initiative, research on the opportunities and challenges of promoting the "the Belt and Road" initiative, research on the path of the "the Belt and Road" initiative, etc. The author of the article has conducted in-depth research on various issues from different perspectives and different concerns. Some of these studies are of great academic value and are of great significance for enriching and developing the theory and practice of the "the Belt and Road" initiative. This paper attempts to sort out the existing literature of the "the Belt and Road" initiative in detail in order to get a clear context and pave the way for subsequent research. This research mainly analyzes the content of the Belt and Road related literature from five perspectives. Research on a community with a shared future, sustainable economic cooperation, political fairness, ecological optimization, and news discourse power. References [6–8] and [14–16] are from the perspective of a community with a shared future, [11–13], [21], and [26–27] are from the perspective of sustainable economic cooperation. And [9–10] and [18] are from the perspective of political fairness. The ecological optimization role of the Belt and Road has been discussed most, [17], [19–25], [28–29] are all the contents of ecological environment development optimization. Finally, [30–34] is a study of the Belt and Road news discourse. The implementation of the Belt and Road Initiative is of great practical significance for the peaceful development of world politics and the transformation and upgrading of China’s economy. Based on the literature content analysis from the above five perspectives, it is expected to provide scientific material support and theoretical reference for the international situation of the Belt and Road.

### 2.1 Studies related to ‘A Community of Shared Future for Mankind’

The idea of Marxist ideology of community is one of the important theoretical sources of the idea of ‘A Community of Shared Future for mankind’ [6]. Zhang [7] also points out that Marxism’s scientific elaboration of the ideology of community has laid the theoretical foundation for the formation of the community of human destiny. The ideology of a community of human destiny is a creative reconstruction of the theoretical leap and paradigm of Marx’s idea of community. Marx’s ideology of community provides the theoretical foundation and methodological basis for a community of human destiny [8].

Lazarus [9] believes that a community is formed voluntarily by people, in which a variety of different people have different strengths and talents, each of whom forms complementary interests with others. Ross [10] links the changing development of the individual to the social whole, arguing that individual claims to specific virtues can only be agreed upon within the notion of the priority of the group.

A large number of scholars consider ‘community’ to refer to a community of interest and argue that the interests of the members are the primary mechanism of connection for the community. Ertz and Leblanc-Proulx [11] argue that community consists of a combination of two basic elements: firstly, an emotion-filled relationship within a certain group of individuals, which is always intertwined and reinforced by each other; secondly, a certain level of commitment to a set of shared values, norms and meanings, as well as a shared history and identity. According to Bouma et al. [12], communities at different levels all have their own interests and can therefore be seen as communities of interest. As globalization continues to develop, a ‘community of cooperation’ has emerged, in which human beings or states are more willing to seek mutually beneficial cooperation in the face of risk, with multipolarity, pluralism, and reciprocal cooperation as its main features [13]. The construction of a ‘community of cooperation’ in the practice dimension and the establishment of a ‘spiritual community’ at the spiritual level constitutes the concept of ‘A Community of Shared Future for Mankind’ [14].

Many scholars hold a positive attitude toward the concept of ‘A Community of Shared Future for Mankind’ and regard it as an innovative, sustainable and holistic concept of development. It is based on equitable and shared development and is a concept with a long-term vision [13]. ‘A Community of Shared Future for Mankind’ is a new concept whose core feature is “win-win cooperation”. It is also a new way of looking at international issues and situations [15].

Research on the connotation of ‘A Community of Shared Future for Mankind’ is more centered on the five dimensions and the human development perspective. According to Zhang [16], the five dimensions refer to political, security, economic, cultural, and ecological, which express a sense of community that aims to promote the survival and development of humanity. However, with the rapid development of ecology in the 20th century and the increasing prominence of global ecological problems, the ecological dimension is the one most often mentioned in the concept of ‘A Community of Shared Future for Mankind’ [17].

Political relations are important institutional connections between the two countries, and friendly political relations are conducive to healthy diplomatic interaction and economic and trade exchanges between the two countries. Lu and others [18] took the political risks and political relations of the countries along "the Belt and Road" as the key factors to study the impact of their political environment on China’s foreign investment, which has important theoretical and practical significance for Chinese enterprises to grasp investment opportunities. Under the guidance of the national "the Belt and Road" strategy, they avoided the political risks of investment and obtained the expected return on investment. China’s direct investment along the "the Belt and Road" roughly starts from neighboring countries and gradually expands to Central and Eastern Europe, with growing strategic influence and radiation. With the continuous fermentation of terrorism, Political violence, armed conflict and anti-government movement, the instability of the global political environment has become increasingly prominent, and the impact of political factors on international trade cannot be ignored.

In addition, in the context of the increasing demand for energy in the international community, the energy issue is no longer just an economic issue, but has gradually evolved into an international political issue, becoming an important tool affecting international and geopolitical relations. Lin and others [19] believed that energy cooperation has always been the focus of international trade along the Belt and Road, and also an important way for countries along the Belt and Road to complement each other’s interests. International energy trade is not only limited to economic activities, but also based on political relations between countries, not only cooperation between enterprises, but also political cooperation between the two countries. Therefore, the promotion of energy cooperation between countries along the Belt and Road is essentially to enhance political mutual trust between the two governments and ensure the political basis of energy cooperation between the two countries.

The proposal of "the Belt and Road" is an external extension of the concept and practice of China’s ecological civilization construction. Based on the experience and achievements of many countries in environmental protection, Huang and others [20] put forward the green development of the "the Belt and Road" and reshaped the development concept of the "the Belt and Road". In the international work of the Belt and Road, the international community actively explores new ways to deal with the inefficient problem of global environmental governance and reduce global carbon emissions.

B&R, one of China’s most prominent international cooperation projects, is considered by many scholars to have a major impact on global trade in the future [18–20]. However, it may lead to permanent environmental degradation.

### 2.2 The studies related to the ecological system along ‘the Belt and Road Initiative’

According to Sarker et al. [21], B&R is divided into three main directions, namely the Northern, Central, and Southeast routes respectively, with essential cities as essential supports. The Northern route starts from the Northwest and Northeast of China and goes directly to Europe via Central Asia and Russia; the Central route starts from the Northwest of China and crosses Central Asia and West Asia before reaching the Persian Gulf and the Mediterranean region; the Southeast route goes from the Southwest of China via Southeast Asia and the Northwest of China via South Asia to the Indian Ocean.

However, environmental problems such as air pollution, soil erosion, and water pollution are still very serious in both China and other countries along B&R [22]. In particular, the western part of China, an essential route for the construction of B&R, has serious land desertification, which plays a pivotal role in constructing B&R [23]. According to Hafeez et al. [24], the Central Asian region also faces problems such as sandstorms, soil erosion, and ecological degradation. Central Asia’s rich mineral resources have attracted the world’s attention. However, due to a lack of financial resources and relatively poor mining technology, the overall exploitation of the resources is not high, and the waste flows directly into rivers or accumulates for a long time, causing serious heavy metal pollution [21]. The study by Saud et al. [23] shows that as the population increases, other CIS countries such as Moldova, Azerbaijan, Armenia, and other areas are logged for heating in winter, massive deforestation and fires have led to a decline in air quality and decreasing forest cover. Also, with the massive exploitation and leakage of oil and gas, animal habitats are threatened, exacerbating biodiversity’s decline and even extinction.

The ecological environment is the material basis and constraint of human existence and development. The current global population boom and resource consumption have created a huge environmental burden. Huang and Yang [25] suggest that China’s political leadership is increasingly concerned about the ecological limits of rapid economic growth due to long-term environmental challenges. Deng et al. [17] believe the project of B&R will have a significant impact on global trade in the future but that it could lead to permanent environmental degradation and that humanity should call for a rigorous system of strategic environmental and social assessment and improved global environmental protection standards.

According to Jiang et al. [26], international environmental organizations have also taken an interest in environmental cooperation in China’s B&R construction and published annual reports with both timely and policy recommendations. Li and Zhu [27] used its technological and networking advantages to conduct a statistical analysis of the natural ecological situation along B&R and proposed that the construction of B&R should focus on the green industry nourishing. In 2013, the United Nations Environment Programme, known as UNEP, also published the ‘Greening the Environment’ report highlighting the unique role it can play in the construction of the green belt and road strategy. For example, the strategy provides support for professional and technical staff and teams and facilitates inclusive green economy financing to promote the development of environmental cooperation between China and countries along B&R [28]. Chen et al. [29] also argue that sustainable development has always been an important issue for the international community, and the green B&R is in line with this trend, incorporating green concepts into all aspects and processes of policy communication, facility links, and trade flows.

### 2.3 The studies related to ‘the Belt and Road Initiative’ news report

B&R is a global initiative proposed by China. In the five years since its launch, the initiative has received positive responses worldwide and has been covered by international and domestic media from all sides [18]. The media is the narrator, disseminator, and interpreter of B&R, meanwhile, it plays a crucial and irreplaceable role in disseminating information, enhancing mutual trust, and building consensus [30]. Xiao et al. [31] point out that as the theoretical studies and projects related to B&R continue to heat up, it has also received extensive attention and coverage from many international media. Researchers are increasingly looking into how the mainstream media in various countries report on B&R in order to listen to the voices of the media in various countries and understand their perceptions of the initiative.

Xiao et al. [31] compared the difference in the construction of media discourse between Chinese and American media. They found that American media uses negative words to describe its political influence, while Chinese media emphasizes the detailed measures of B&R with positive words. Zhang & Wu [32] also found the similarity while exploring the difference in the construction of media discourse between Chinese and English. Although the UK media considers B&R as the most influencing policy in the 21^st^ century, it still describes China as an autarchy that wants to dominate the world. In contrast, Chinese mainstream media tries to construct a country that adheres to peace, and the advantages of B&R. Yu’s [33] study points out that regional media is of vital importance in the publicity of regional image; therefore, each region or country along B&R should utilize media to advertise their distribution and advantages in B&R. Afzaal et al. [34] suggest that China and America have different public diplomacy media construction, while America put more focus on political meaning.

To sum up, many existing studies on the media discourse on B&R, but very few researchers have paid attention to and studied the ecological issues accompanying the media coverage of B&R and its discursive construction. Compared with normal media discourse analysis on B&R, it is obvious that how to report the current environmental and ecological issues along it is much more worth exploring.

## 3 Methodology

### 3.1 Sampling

In this study, twenty news stories related to environmental issues from B&R countries were randomly selected from Google for in-depth research and analysis. These reports covered the ‘Belt and Road Environmental Cooperation’ from July 2021 to August 2022. The literature reviewed and analyzed in this study involves the international environment and Ideology#Political ideologies in the Belt and Road, and develops the sustainability of economic cooperation, aid to poor countries, ecological environment protection, energy consumption and other aspects in the theory of a community of shared future.

### 3.2 Theoretical framework

**Fig 1:**
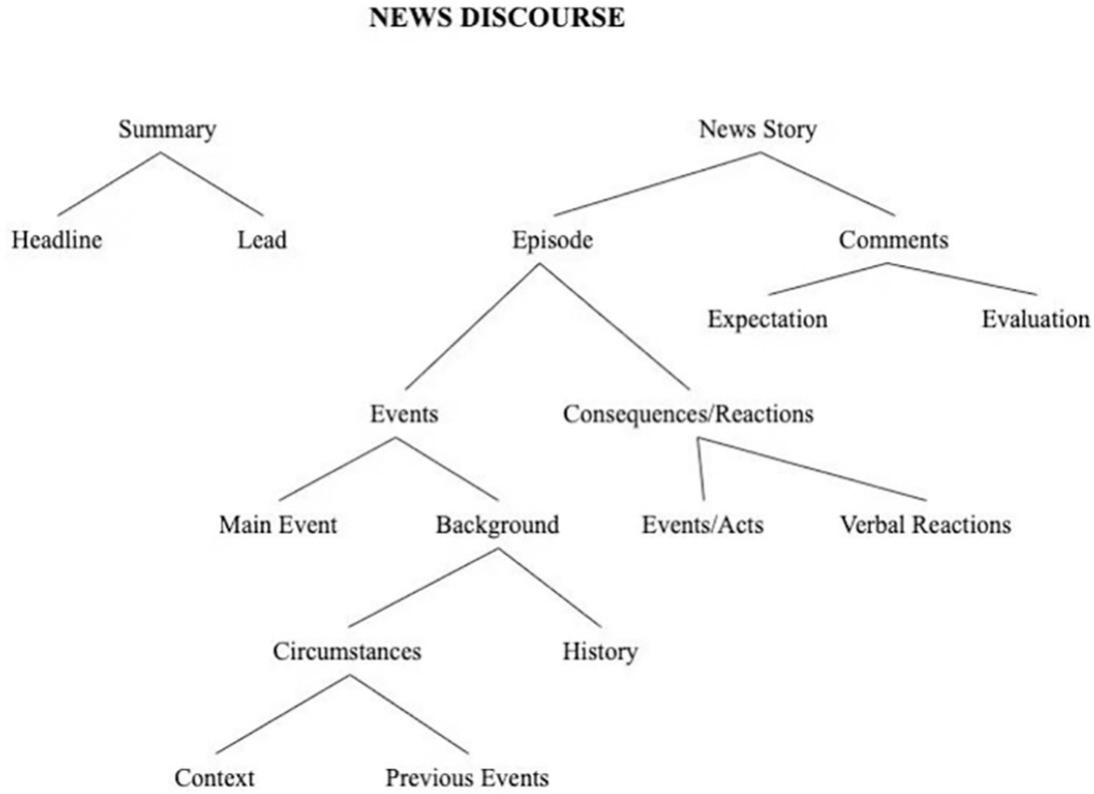
Hypothetical structure of news schema.

News, as a discourse text, is constructed through spoken and written information conveyed in a linguistic descriptive system. Van Dijk’s [5] theory of discourse analysis, especially the structural discourse analysis of texts, is applicable to the analysis of news discourse. The approach to news discourse analysis suggests that news production is a discourse practice that directly affects the ‘structure’ of society as a whole rather than merely the attitudes of a single audience, the perceptions of society, and the setting of the public agenda. While previous studies of news discourse have generally focused on the social, cultural, or ideological aspects of news production and rarely on the news text itself. Van Dijk [5] proposes that the analysis of news discourse should include not only an analysis of context but also a richer type of textual analysis. This means that news discourse is analyzed from both textual and contextual perspectives. The textual perspective describes the structure of discourse at each level. The contextual perspective examines the description of these structures in relation to various features of the context, such as cognitive processes, reproduction, and socio-cultural factors. On this basis, Van Dijk proposes a framework for the discourse analysis of news texts (Fig 1).

While thematic and schematic analyses are macro-analyses. While the micro-structure of news discourse includes the semantic structure.

Van Dijk [5] defines style as the manifestation or signification of the social characteristics of the speaker and the specific features of the social culture of the context in which he is speaking. The choice of a particular variant results in a certain stylistic characteristic. For example, style is the expression of the same meaning in different ways. The style of journalism is also controlled by the subject matter of the news discourse, its printed form, publicity, institutional impersonality, and formality. The demands and requirements of production all contribute to the complex and fixed stylistic features that are evident in newspaper journalism.

The rhetoric of discourse is also about how it is expressed. The rhetoric of news is not limited to the use of common rhetorical devices; rather, it includes strategic devices used to increase the truthfulness, rationality, correctness, accuracy, and credibility of news stories. These strategies can include the extensive use of data, the selection of sources, the selective choice of viewpoints, and so on.

This study takes Van Dijk’s framework of journalistic discourse analysis as the theoretical framework and uses the news text of the B&R environment-related report from a variety of countries to conduct a systematic and in-depth analysis of the journalistic discourse from the text.

## 4 Results and discussion

### 4.1 The macro-structure of reports

#### 4.1.1 Theme structure

The textual perspective is a description of the structure of the discourse at various levels. This chapter analyses the textual structure of the Belt and Road Initiative at both macro and micro levels, based on Van Dijk’s [5] discourse analysis theory. Due to space constraints, it is impossible to conduct a statistical analysis of all the reports. Therefore, a case study approach has been adopted, and specific texts have been selected for analysis.

One way of representing the macro-thematic structure of a text is a tree diagram. A story usually contains several themes, which are made up of propositions. Propositions are the smallest, independent units of meaning in language and thought.

**Fig 2:**
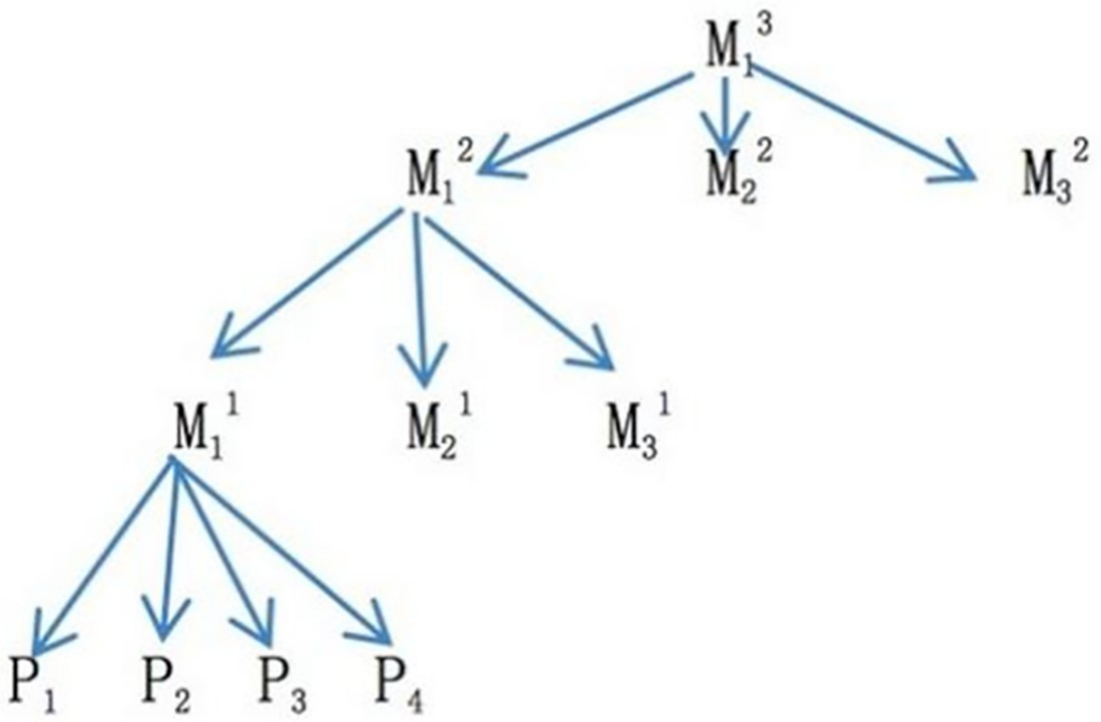
An overall diagram of the semantic macro-structure of the text.

The propositions are the smallest, independent units of meaning in language and thought. Fig 2 shows that propositions P_1_, P_2_, P_3_, and P_4_ constitute the first-level theme M_1_^1^. Themes M_1_^1^, M_2_^1^, and M_3_^1^ form the second-level theme M_1_^2^. The second-level themes M_1_^2^, M_2_^2^, and M_3_^2^ form the third-level theme M_1_^3^. However, a story may contain more than one topic. In order to gain a multi-level and multi-faceted understanding of the thematic structure of mainstream newspapers on B&R, two news stories were randomly selected for analysis.

1) News headline: *China*, *Africa pursue green development under BRI*, *improving Africans’ livelihood*

**Fig 3:**
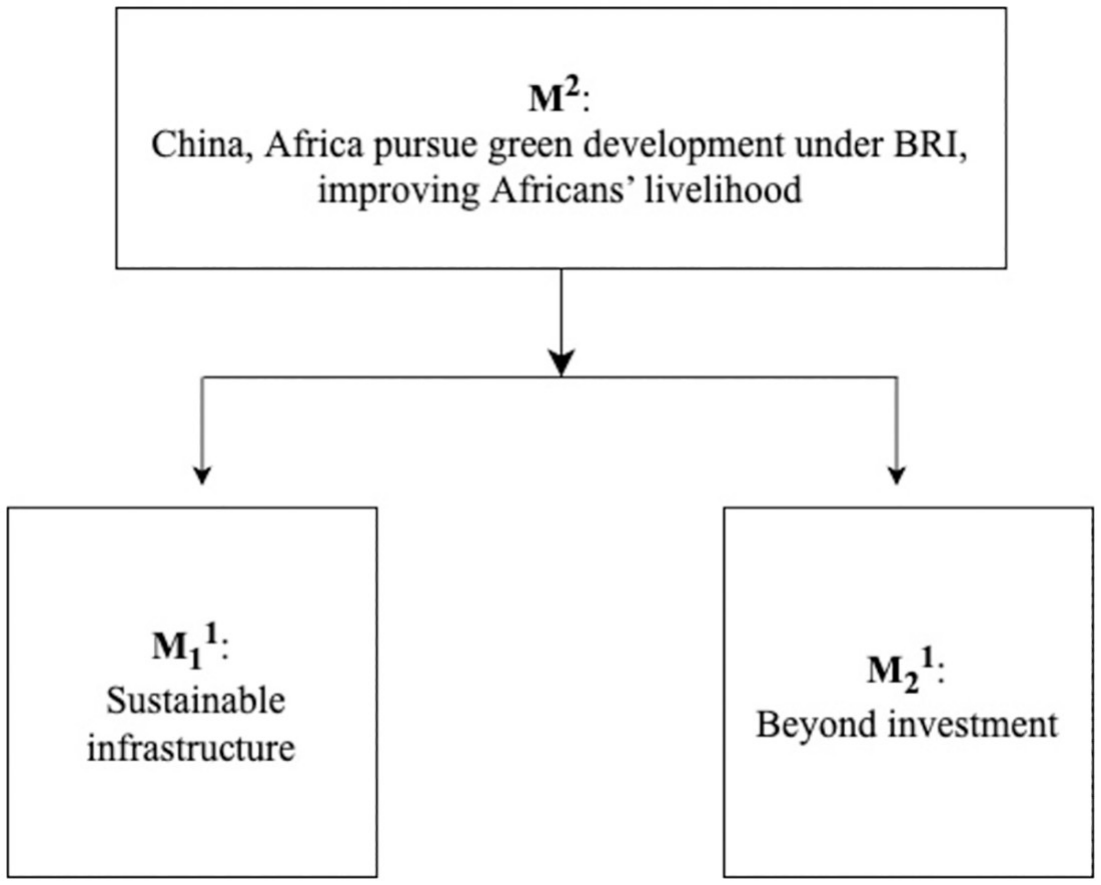
The theme structure of ‘China, Africa pursue green development under BRI, improving Africans’ livelihood’.

This news story was published on the website of Global Times on 2^nd^ December 2021. In the report, the headline ‘China, Africa pursue green development under BRI, improving Africans’ livelihood’ directly and distinctly explains the theme of the report, which we have labeled its headline as M^2^ based on Van Dijk’s [5] theory (Fig 3). M^2^ is divided into two macro-themes: “Sustainable infrastructure” (M_1_^1^) and “Beyond investment” (M_2_^1^). M_1_^1^ points out that cooperation between China and Africa should be geared towards the active development of renewable energy and the continued improvement of sustainable development, and M_2_^1^ explains how Chinese companies’ investments in Africa are aimed at improving the livelihoods of local people and economic development.

2) News headline: *Xi looks to greener growth across the globe*

**Fig 4:**
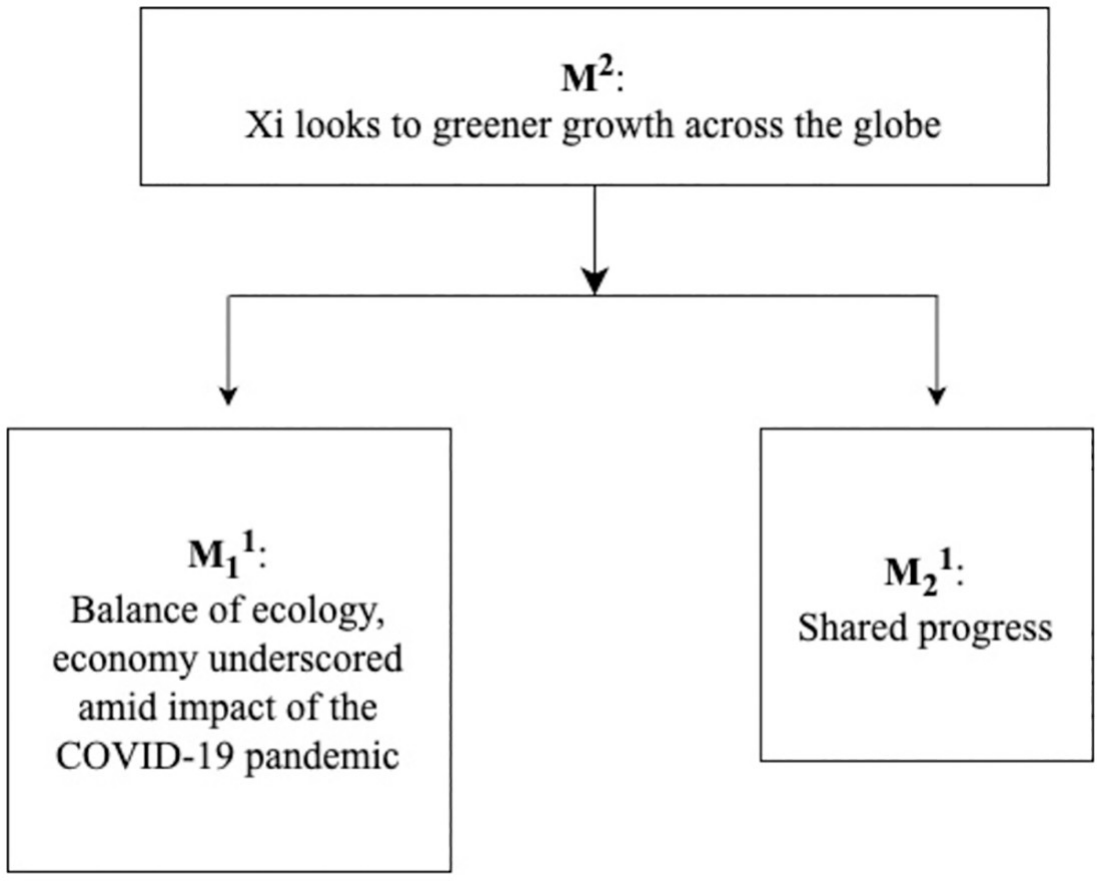
The theme structure of ‘Xi looks to greener growth across the globe’.

This news story was published on the website of China Daily on 24^th^ December 2021. In the report, the headline ‘Xi looks to greener growth across the globe’ directly and distinctly explains the theme of the report, which we have labeled its headline as M^2^ based on Van Dijk’s [5] theory (Fig 4). M^2^ is divided into two macro-themes: “Balance of ecology, economy underscored amid impact of the COVID-19 pandemic” (M_1_^1^) and “Shared progress” (M_2_^1^). M_1_^1^ carries on the spirit of the balance between economic development and the environment among countries, and M_2_^1^ advocates the importance of global environmental cooperation.

Through the analysis of macro themes, it can be seen that the themes of these reports on environmental cooperation in B&R countries are evident. Firstly, the title of the report on environmental cooperation represents the theme; secondly, there is a clear hierarchical relationship between the stories on environmental cooperation in B&R countries; thirdly, through the presentation of specific issues related to environmental cooperation, the news from each country is not only reported as objective facts but also has a corresponding construction of the news context and meaning of communication.

#### 4.1.2 The news schema analysis

**Fig 5:**
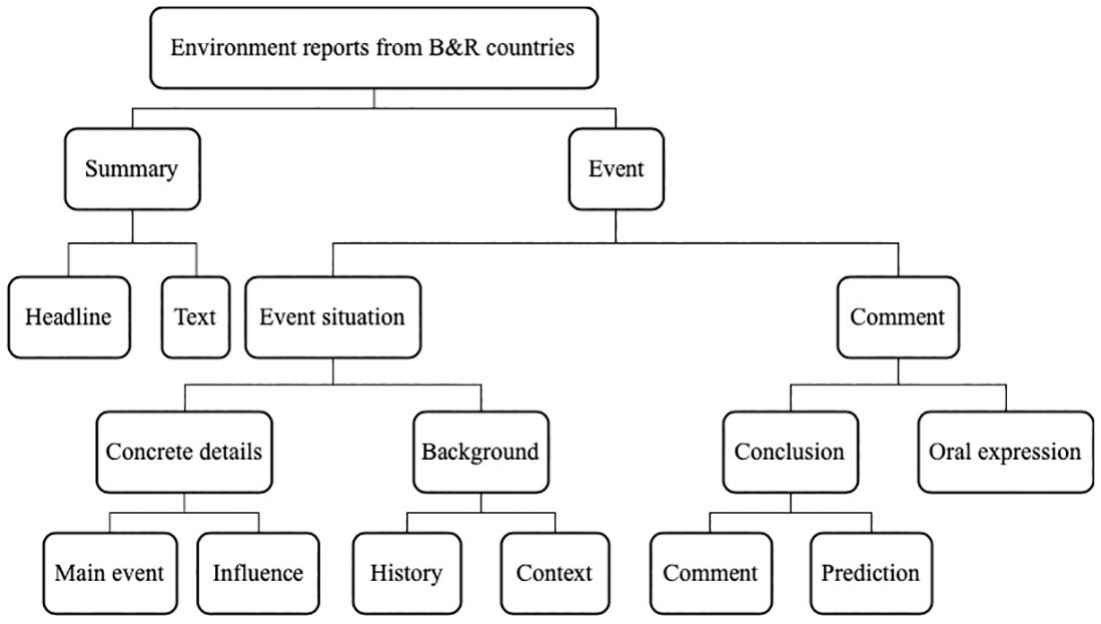
The general news report schema of environment report from B&R countries.

The most important part of the macro-structural analysis of environment cooperation report from B&R countries is to analyze the global structure of its discourse, exploring the textual form of its discourse and the process of its production and interpretation in order to draw up a linking news diagram. After a comprehensive review of the research sample, the environment cooperation report from B&R countries can be represented (Fig 5).

The analysis of news diagrams on the same theme in these mainstream media reports from B&R countries shows that:

1. Where the subject matter is the same, the structure of the news diagrams is also generally consistent. The news schema of the environmental cooperation reports from B&R countries is consistent with the hypothetical news schema proposed by Van Dijk [5] and has an even more complete structure.
2. Most reports begin with a straightforward statement of the facts of the story. The headline and introduction in the overview are usually completed in the first paragraph.
3. The main event may be only one or more within a story. The commentary section is not usually direct but is expressed in the words of others, either experts or ordinary citizens.

### 4.2 The micro-structure analysis

The above analysis of the macro schema of environmental cooperation report from B&R countries is based on Van Dijk’s [5] theory of journalistic discourse analysis, which also requires an examination of the micro perspective. Analyzing micro-structures helps to understand the process of constructing themes in specific news texts. Specifically, the study can be respectively conducted regarding the implicit meaning of news discourse and rhetoric.

#### 4.2.1 The implicit meanings in news discourse

News has many hidden meanings in the text. The wording and implied meanings in news stories reflect the journalist’s assessment from his/her point of view and position. Van Dijk [5] argues that the choice of words in a news report is a more direct expression of the hidden ideology behind the news report.

From the above macro-structural analysis, evidently, the headline generally represents the central event of the environmental cooperation report from B&R countries. Analysis of the implied meaning of the headlines in mainstream newspapers in B&R countries is, therefore, the focus.

Many headlines in mainstream newspapers from B&R countries contain different words or phrases. For this reason, the words that appear in the headlines are counted according to their lexical categories (Table 1). Words are classified according to their grammatical function and morphology. Words are classified as verbs, adjectives, adjectival verbs, nouns, adverbs, conjunctions, conjunctions, exclamations, auxiliary verbs, auxiliary words, etc. With the exception of verbs, adjectives, nouns, and adverbs, all the other words are modifiers and have no real meaning, so they are not counted. However, as adverbs hardly occur, they are not listed separately here either.

**Table 1:** The lexis used in headlines.

Verbs	achieve, appealed, avoid, be, continues, develop, fence, goes, hasten, improving, lead, looks, looms, plans, pursue, reduce, sees, slashes, start, taken, wrapped
Nouns	Africa, Africans’, Antarctic, Belarus, Belovezhskaya, BRI, carbon, care, catastrophe, changes, China, climate, construction, council, country, crisis, development, ecology, efforts, emissions, energy, environment, Europe, footprint, future, gas, greenhouse, growth, guideline, livelihood, iceberg, look, neutrality, planet’s, power, progress, projects, protection, rages, reckoning, resources, revolution, Russia, Russia’s, technologies, throttle, transition, vision, Xi
Adjectives	due, economic, emerging, environmental, full, global, green, important, looming, overseas, planetary, renewable, updated, war

Nouns, to a certain extent, represent ‘what is’. This means that the noun in the story’s title indicates the environmental problem or measure. On the other hand, the verb indicates the action being performed and, to some extent, what is being ‘done’. The use of these words indicates that environmental problems and cooperation are valued by B&R countries. Adjectives are mainly used to modify nouns and to express the nature or state of a person or thing. Adjectives often have a positive or negative connotation, and as journalism is characterized by truthfulness and objectivity, adjectives rarely appear in the headlines; in mainstream newspapers on B&R’s environmental problems and cooperation, they are used to highlight the global and economic nature of environmental issues.

The analysis shows that B&R countries have chosen to use positive words, both verbs and nouns, and have avoided emotive words, which indicates they are seeking cooperation. For example, the words ‘global’, ‘appeal’, and ‘achieve’ align with the core belief of ‘A Community of Shared Future for Mankind’. The reporters use the simplest terms to describe existing environmental issues and measures, thusly, readers can understand them at a glance. These choices of words indicate the hidden meanings in the coverage of environmental issues and cooperation in B&R countries’ newspapers.

#### 4.2.2 Rhetoric analysis

The use of rhetorical devices and structures in journalism depends on the communication’s objective and intended effect. In other words, rhetorical devices are used to persuade readers to accept the article’s point of view. The rhetorical devices Van Dijk [5] outlined mainly include sources, quotations, and the adoption of numbers.

The B&R countries’ newspapers have been quoting direct quotes from leaders on environmental protection initiatives. For example, a direct quote from the Chinese vice-minister: “In recent years, the country’s investment in renewable energy projects in countries and regions involved in the BRI has kept growing. Also, The law still permits building major objects (pipelines or highways) through federal OOPTN with no environmental review”, said Yulia Davydova, a spokesperson for environmental group Greenpeace’s Russian branch, who was directly quoted. Besides, indirect quotations are also used, for instance, ‘after so many years of the pressure of sanctions and demonization of Russia, they still think that they are entitled to demand additional gas supplies from Gazprom’. The complementarity of direct and indirect quotations avoids monotony and increases the story’s readability.

The rhetoric of news discourse also strongly suggests its truth through the precision implied by the exact numerical lock. As Van Dijk [5] argues, it is not the precision of the numbers that really matter but the facts that are expressed through them.

A large amount of data was also used in B&R countries’ news. The data types are time-date and percentage.

1) Time-date

Each report indicates the timing of the events mentioned in the article.

For example:

*“Compared to 1990*, *greenhouse gas emissions in Russia decreased from 3*.*1 billion tons of CO2 equivalent to 1*.*6 billion”;*

*“The 2021 Leaders’ Climate Summit was held in April*. *President Vladimir Putin said during his speech at the summit”;*

*“Kenneth Cheng*, *managing director at Accenture*, *said Chinese utility companies and power companies have built up sufficient experience for digital solutions in the past few years*, *and the 14th Five-Year Plan period (2021–25) will witness increasing adoption of data to further facilitate the country’s power sector transition”*.

2) Percentage

Most of the reports on environmental issues from B&R countries mainly used percentages to explain the proportion of the speed and space.

For example:

*“In particular*, *a law passed last month made it easier for businesses to embark on major construction projects in Specially Protected Natural Territories (OOPT)*, *which cover some 12*.*5% of Russian territory”;*

*“The Russian economy may contract by more than 10% this year*, *according to predictions by the World Bank”*;

*“Reports on environmental issues from B&R countries mainly used percentage to explain the proportion of the speed*, *space”*.

## 5 Conclusion

This study presents a macro and micro analysis of the coverage of environmental cooperation on reports of environmental issues in B&R countries. At the macro level, the headline contains the main theme, which is a brief and concise statement of the central content of the story. Where the theme is the same, the diagram structure of the story is also generally consistent, with the evaluation of the article generally appearing at the end in the words of others. At the micro level, the choice of words in the headlines indicates a neutral and positive attitude towards environmental cooperation in the national media. Most B&R countries adhere to ‘A Community of Shared Future for Mankind’, realize the environmental problems and seek international cooperation.

The fact that many countries do not have official English-language newspapers impacts the analysis of the text and the conclusions drawn from this article due to the difficulty of obtaining a sample. In addition, the sample size chosen for this study was relatively small, so the findings of this article are not universal. This study has reference significance for the green development concept to promote the "the Belt and Road" towards a higher stage and a higher level of development. This study believes that the role of the state, enterprises and other different subjects should be played at the same time, and scientific research should grasp the key on the basis of grasping the overall situation.

With the development of B&R, there will be more and more cooperation jumping in. Therefore, in addition to environmental cooperation, the mainstream media will also cover many other aspects of cooperation. In future studies, B&R’s economic and political cooperation may also become a hot topic for coverage. Subsequent relevant research and practice promotion will provide a more professional and systematic theoretical basis, detailed empirical data and case studies for the high-quality development of the "the Belt and Road", enrich the research of this paper, enrich the new model of high-quality development of the green "the Belt and Road", and hope that the research in this area can make a breakthrough in combination with the new requirements of high-quality development.

## References

[1] Turcsányi RQ. China and the frustrated region: Central and Eastern Europe’s repeating troubles with great powers. China Report, 2020;56(1):60–77. doi: 10.1177/0009445519895626

[2] Vörös Z, Somsack P. Laos and the belt and road initiative: An interconnector helping the Chinese needs? Foreign Policy Review, 2020;13:24–38. doi: 10.47706/KKIFPR.2020.13.24-38

[3] Hurley J, Morris S, Portelance G. Examining the debt implications of the Belt and Road Initiative from a policy perspective. Journal of Infrastructure, Policy and Development, 2019, 3(1): 139–175. doi: http%3A//dx.doi.org/10.24294/jipd.v3i1.1123

[4] Tarrósy I. China’s belt and road initiative in Africa, debt risk and new dependency: The case of Ethiopia. African Studies Quarterly, 2020;19(3–4):8–28. https://asq.africa.ufl.edu/files/V19i3-4a2.pdf

[5] Van Dijk TA. News as Discourse. Hillsdale, NJ: Lawrence Erlbaum Associates, 1988.

[6] Shen Z. The construction of human community with A Shared Future for Mankind from the perspective of Marxist communication theory. Advances in Philosophy, 2022;11(4):555–561. doi: 10.12677/ACPP.2022.114099

[7] Zhang H. A community of shared future for mankind–the contemporary development of the social foundations theory of international law. Social Sciences in China, 2019;40(1):186–202. doi: 10.1080/02529203.2019.1556489

[8] Zhao X. In pursuit of a community of shared future: China’s global activism in perspective. China Quarterly of International Strategic Studies, 2018;4(1):23–37. doi: 10.1142/S2377740018500082

[9] Lazarus M. Politics in the conflicts of modernity: Aristotelian and Marxist. International Critical Thought, 2019;9(3):463–479. doi: 10.1080/21598282.2019.1647549

[10] Ross EW. Humanizing critical pedagogy: What kind of teachers? What kind of citizenship? What kind of future? Review of Education, Pedagogy, and Cultural Studies, 2018;40(5):371–389. doi: 10.1080/10714413.2019.1570792

[11] Ertz M, Leblanc-Proulx S. Sustainability in the collaborative economy: A bibliometric analysis reveals emerging interest. Journal of Cleaner Production, 2018;196:1073–1085. doi: 10.1016/j.jclepro.2018.06.095

[12] Bouma J, Montanarella L, Evanylo G. The challenge for the soil science community to contribute to the implementation of the UN Sustainable Development Goals. Soil Use and Management, 2019;35(4):538–546. doi: 10.1111/sum.12518

[13] Krings A, Schusler T. Equity in sustainable development: Community responses to environmental gentrification. International Journal of Social Welfare, 2020;29(4):321–334. doi: 10.1111/ijsw.12425

[14] Xu F, Su J. Shaping “A Community of Shared Future for Mankind”: New elements of general assembly resolution 72/250 on further practical measures for the PAROS. Space Policy, 2018;44–45:57–62. doi: 10.1016/j.spacepol.2018.04.002

[15] Pan J, Chen C, Yang Y. Building a global community of shared future free from poverty. Global Health Journal, 2021;5(3):113–115. doi: 10.1016/j.glohj.2021.08.001 34580618 PMC8457890

[16] Zhang D. The concept of ‘Community of Common Destiny’ in China’s diplomacy: Meaning, motives and implications. Asia & The Pacific Policy Studies, 2018;5(2):196–207. doi: 10.1002/app5.231

[17] Deng Y, Randall J, Ye F. Island ecological restoration and management practices based on nature: Conference report. Marine Policy, 2022;143:105188. https://doi.org/10.1016%2Fj.marpol.2022.105188 35945916 10.1016/j.marpol.2022.105188 PMC9352228

[18] Lu Y, Gu W, Zeng K. Does the belt and road initiative promote bilateral political relations?. China & World Economy, 2021;29(5):57–83. doi: 10.1111/cwe.12387

[19] Lin B, Bega F. China’s belt & road initiative coal power cooperation: Transitioning toward low-carbon development. Energy Policy, 2021;156:112438. doi: 10.1016/j.enpol.2021.112438

[20] Huang M, Li S. The analysis of the impact of the Belt and Road initiative on the green development of participating countries. Science of the Total Environment, 2020;722:137869. doi: 10.1016/j.scitotenv.2020.137869 32208256

[21] Sarker M, Hossin M, Yin X, Sarkar M. One Belt One Road Initiative of China: Implication for future of global development. Modern Economy, 2018;9(4):623–638. doi: 10.4236/me.2018.94040

[22] Sun Q, Geng Y, Ma F, Wang C, Wang B, Wang X, et al. Spatial-temporal evolution and factor decomposition for ecological pressure of carbon footprint in the One Belt and One Road. Sustainability, 2018;10(9):3107. doi: 10.3390/su10093107

[23] Saud S, Chen S, Haseeb A, Sumayya. The role of financial development and globalization in the environment: Accounting ecological footprint indicators for selected one-belt-one-road initiative countries. Journal of Cleaner Production, 2020;250:119518. doi: 10.1016/j.jclepro.2019.119518

[24] Hafeez M, Yuan C, Khelfaoui I, Sultan Musaad OA, Waqas Akbar M, Jie L. Evaluating the energy consumption inequalities in the One Belt and One Road region: Implications for the environment. Energies, 2019;12(7):1358. doi: 10.3390/en12071358

[25] Huang J, Yang J. Beyond under the dome: an environmental documentary amplified public risk perception about air pollution in China. Journal of Risk Research, 2019;23(2):227–241. doi: 10.1080/13669877.2019.1569090

[26] Jiang Q, Ma X, Wang Y. How does the one belt one road initiative affect the green economic growth? Energy Economics, 2021;101:105429. doi: 10.1016/j.eneco.2021.105429

[27] Li Y, Zhu X. The 2030 agenda for sustainable development and China’s belt and road initiative in Latin America and the Caribbean. Sustainability, 2019;11(8):2297. doi: 10.3390/su11082297

[28] Ascensão F, Fahrig L, Clevenger AP, et al. Environmental challenges for the Belt and Road Initiative. Nature Sustainability, 2018, 1(5): 206–209. doi: 10.1038/s41893-018-0059-3

[29] Chen J, Rojniruttikul N, Kun L, Ullah S. Management of green economic infrastructure and environmental sustainability in One Belt and Road Enitiative Economies. Environmental Science and Pollution Research, 2022;29(24):36326–36336. doi: 10.1007/s11356-021-18054-5 35060037

[30] Karakaya S, Glazier R. Media, information, and political participation: The importance of online news sources in the absence of a free press. Journal of Information Technology & Politics, 2019;16(3):290–306. doi: 10.1080/19331681.2019.1645784

[31] Xiao Y, Li Y, Hu J. Construction of the Belt and Road Initiative in Chinese and American media: A critical discourse analysis based on self-built corpora. International Journal of English Linguistics, 2019;9(3):68–77. doi: 10.5539/ijel.v9n3p68

[32] Zhang L, Wu D. Media representations of China: A comparison of China Daily and Financial Times in reporting on the Belt and Road Initiative. Critical Arts, 2017;31(6):29–43. doi: 10.1080/02560046.2017.1408132

[33] Yu J. Research on media image of Xi’an construction in the discourse of “One Belt One Road”. In W. Striełkowski (Ed.), Proceedings of the First China Xijing Intelligent Media Forum (CXIMF 2020). Atlantis Press. 2020, 10.2991/assehr.k.201102.002.

[34] Afzaal M, Zhang C, Chishti M. Comrades or contenders: A corpus-based study of China’s Belt and Road in US diplomatic discourse. Asian Journal of Comparative Politics, 2022;7(3):684–702. doi: 10.1177/20578911211069709

